# Conditional Proximity Labeling in Complex Biological Systems: Design Principles, Emerging Platforms, and Applications

**DOI:** 10.3390/ijms27209284

**Published:** 2026-10-19

**Authors:** Jia-Ming Yang, Meng-Die Zhu, Shu-Lin Liu, Long Yang, Zhi-Gang Wang

**Affiliations:** 1 State Key Laboratory of Medicinal Chemical Biology, School of Medicine and Research Centre for Analytical Sciences, College of Chemistry, Nankai University, Tianjin 300071, China; eva1dzs.dr.yang@outlook.com (J.-M.Y.); shulin.liu@nankai.edu.cn (S.-L.L.); 2 School of Integrative Medicine, Tianjin University of Traditional Chinese Medicine, Tianjin 301617, China; mengdiezhu@mail.nankai.edu.cn

**Keywords:** proximity labeling, conditional proximity labeling, split enzymes, intercellular interactions, light-controlled proximity labeling, orthogonal labeling, spatial proteomics

## Abstract

Proximity labeling (PL) enables covalent capture of biomolecules near a defined target in living systems, providing a route to map molecular proximity in situ. Conventional PL usually relies on a single targeting cue and may therefore lack the selectivity required to resolve defined molecular events or dynamic processes in complex biological systems. Conditional PL addresses this limitation by coupling labeling activity to additional inputs, including interaction-dependent enzyme reconstitution, localization of catalytic modules at cell–cell interfaces, light-regulated catalysis, and multiple orthogonal labeling reactions. These designs allow labeling to respond to specified spatial, temporal, or molecular conditions. We group conditional PL into four designs—split complementation, interface localization, light control, and multiple labeling—based on what activates labeling and what the labels reveal. We examine their use in studying protein complexes, trafficking, and intercellular communication. We also consider what each strategy encodes, the experimental boundaries of the resulting proximity information, and trade-offs among selectivity, labeling efficiency, temporal control, and compatibility with native biological contexts. Remaining challenges include expanding catalyst and probe diversity, improving in vivo compatibility, and integrating conditional PL with single-cell analysis, spatial omics, and high-resolution imaging.

## 1. Introduction

The functions of eukaryotic cells depend on dynamic networks of interactions among biomolecules. Proteins, as the principal executors of cellular functions, rarely act in isolation: enzymes assemble into multicomponent complexes, receptors cooperate with signaling partners, and scaffold proteins organize higher-order structures [1]. These protein–protein interactions (PPIs), together with transient and spatially restricted molecular contacts, underpin cell signaling, material transport, and homeostatic regulation [2]. Accurately mapping the relevant molecular interactions and proximity relationships in their native context has therefore become a central task in understanding cellular function and disease mechanisms [3,4,5]. Conventional biochemical methods, including affinity purification coupled with mass spectrometry (AP–MS) [6,7,8], co-immunoprecipitation [9], and yeast two-hybrid assays [10], have provided foundational insights into interactomes. Their dependence on cell lysis and complex stability, however, substantially limits the capture of transient, low-affinity, or spatially restricted interactions [11].

Proximity labeling (PL) offers a distinct route for directly probing the spatial proximity of biomolecules in intact cells [12]. In this approach, an engineered enzyme is genetically fused to a protein of interest, or a photocatalyst is anchored to a target region through chemical or antibody conjugation. The catalyst then generates reactive species that covalently label nearby biomolecules [11,12,13]. Labeled products are affinity-enriched and identified by mass spectrometry or high-throughput sequencing. Since the establishment of the first enzyme-based PL system, the field has expanded to include platforms based on enzymatic, photocatalytic, and chemically activated triggers [12,14,15]. By preserving spatial-proximity evidence generated by low-affinity or transient molecular contacts through covalent labeling, PL has been widely applied to map target-centered proximal proteomes [16,17,18,19]. Nevertheless, PL results primarily report spatial proximity; direct physical interactions still require appropriate controls and orthogonal validation.

However, PL methods that generally depend on a single biological level of spatial targeting—that is, fusion of the labeling enzyme to one bait protein or one subcellular localization signal—increasingly reveal fundamental limitations when applied to defined spatial relationships and condition-dependent events in complex biological systems [20]. Tasks such as distinguishing a specific subpopulation within a mixed cell population or profiling proteins proximal to a defined multicomponent complex often require selectivity beyond that provided by a single spatial cue [20,21]. To address this limitation, a series of conditional PL strategies have been developed by adding interaction-dependent enzyme reconstitution [22,23,24,25], contact-dependent interface labeling [26,27,28], light-based regulation [29,30], or combinations of multiple labeling reactions [31,32,33,34]. These additional inputs allow labeling events to encode more dimensions of spatial and dynamic information and broaden the use of PL in complex biological systems. These approaches extend PL to the study of molecular complexes, protein trafficking, and intercellular communication [21,31]. This review organizes platforms by what activates labeling and what the resulting labels reveal: proximity around a molecular event, cell contact, an illuminated neighborhood, or spatial origin and movement between compartments. This links each design to the biological questions it can address and the limits of its readout. Table 1 compares representative platforms to guide method selection.

**Table 1:** Comparison of representative conditional proximity-labeling platforms. MS, mass spectrometry. Times refer to labeling or signal detection, not the full workflow. Where no numerical time is given, the mode of temporal control is described. Labeling range depends on the probe and experimental conditions. An em dash indicates that no quantitative value is listed here, not that the original study performed no measurement. Multiple labeling across different compartments is not summarized by a single radius.

Platform	Control Input	Catalyst/Enzyme	Probe/Substrate	Approximate Range	Labeling Time or Temporal Control	Readout and Use	Requirements/Limitations
Split-BioID [22]	Target association enables complementation	Complementary BirA* fragments	Biotin	—	Several hours	MS; proteins near a complex	Slow labeling limits capture of transient events.
Split-TurboID [23]	Fragment proximity and complementation	Complementary TurboID fragments	Biotin	—	Signal detected at 30 min in an induced-complementation assay; longer for proteomics	MS; proteins near organelle contacts	Requires targeting and complementation of both fragments.
FucoID [28]	Direct cell contact	Cell-surface fucosyltransferase	GDP–fucose–biotin	—	Protocol-dependent	Cell biotin signal; contacting cells and antigen-reactive T cells	Requires surface enzyme and accessible acceptor glycans; does not identify receptor– ligand pairs.
LOV-Turbo [29]	Blue-light-induced conformational change	LOV-regulated TurboID	Biotin	—	Light controls labeling onset and termination	MS; protein trafficking during stress	Requires fusion-enzyme expression and control of photoactivation.
RinID [35]	Blue-light activation	miniSOG	Amine probes	—	Minute-scale illumination	MS; nearby proteins	Requires photocatalyst expression; probe and illumination conditions affect labeling.
LITag [36]	Blue-light activation	LOV* flavoprotein	Biotin–phenol or biotin–aniline	—	Seconds of illumination in live-cell experiments	MS; rapid labeling of nearby proteins	Requires LOV* expression; short pulses depend on the reported illumination conditions.
Lantern [37]	Blue-light activation	Lantern, evolved from miniSOG	Representative: propargylamine (CAP-seq); 3-ethynylaniline (CAP-MS); biotin-conjugated primary amine (CAP-CELL)	—	Sub-minute RNA and protein labeling in separate assays	RNA sequencing (CAP-seq), MS (CAP-MS), or cell-surface biotin tagging (CAP-CELL); nearby RNA, proteins, or selected cells	Probe and illumination conditions differ between analytes.
MultiMap [30,38]	Catalyst targeting and illumination	Eosin Y	Diazirine, aryl azide, or phenol	Probe-dependent; no single radius listed	Light-defined labeling window	MS; EGFR neighborhoods and intercellular synapses	Probe selection changes the labeling chemistry and range.
CAT-Tissue [39]	Nanobody targeting and deep-red light	Nanobody–chlorin e6	Biotin–aniline	—	Light-defined labeling window	Cell labeling with bulk RNA sequencing; tumor-proximal versus distal lymphocytes	Requires nanobody targeting and tissue illumination.
TransitID [32]	Sequential labeling in source and destination compartments	TurboID, then APEX2	Biotin, then alkyne–phenol	No single radius applies	Separate labeling steps; trafficking-assay-dependent	MS of dual-tagged proteins; intercompartmental trafficking	Requires targeting of both enzymes and detection of both tags.
OrthoID [33]	Orthogonal labeling in two organelles	TurboID and APEX2	Biotin and phenol- conjugated adamantane	No single radius applies	Set by the two enzyme-labeling protocols	MS; organelle origin and candidate contact-site proteins	Requires enzyme targeting and separate tag recognition and enrichment.

## 2. Conditional Proximity Labeling Mediated by Split Complementation

Split proximity labeling is a well-established implementation of logic-gated PL. A PL enzyme is divided into two inactive fragments, each fused to a different target molecule. Only when the targets interact or approach within a distance that permits fragment complementation are the two fragments brought together and enzyme activity restored, initiating PL in the region surrounding the molecular event [22,23,40]. Conceptually, split complementation extends the protein-fragment complementation assay (PCA) to PL [22,24]. Whereas conventional PCA uses fluorescence or reporter-gene activity as its readout, split PL produces covalently labeled products that can be affinity-enriched and identified by mass spectrometry. Split PL can therefore validate binary PPIs and screen candidate proteins located near a defined molecular complex or subcellular region.

**Figure 1:**
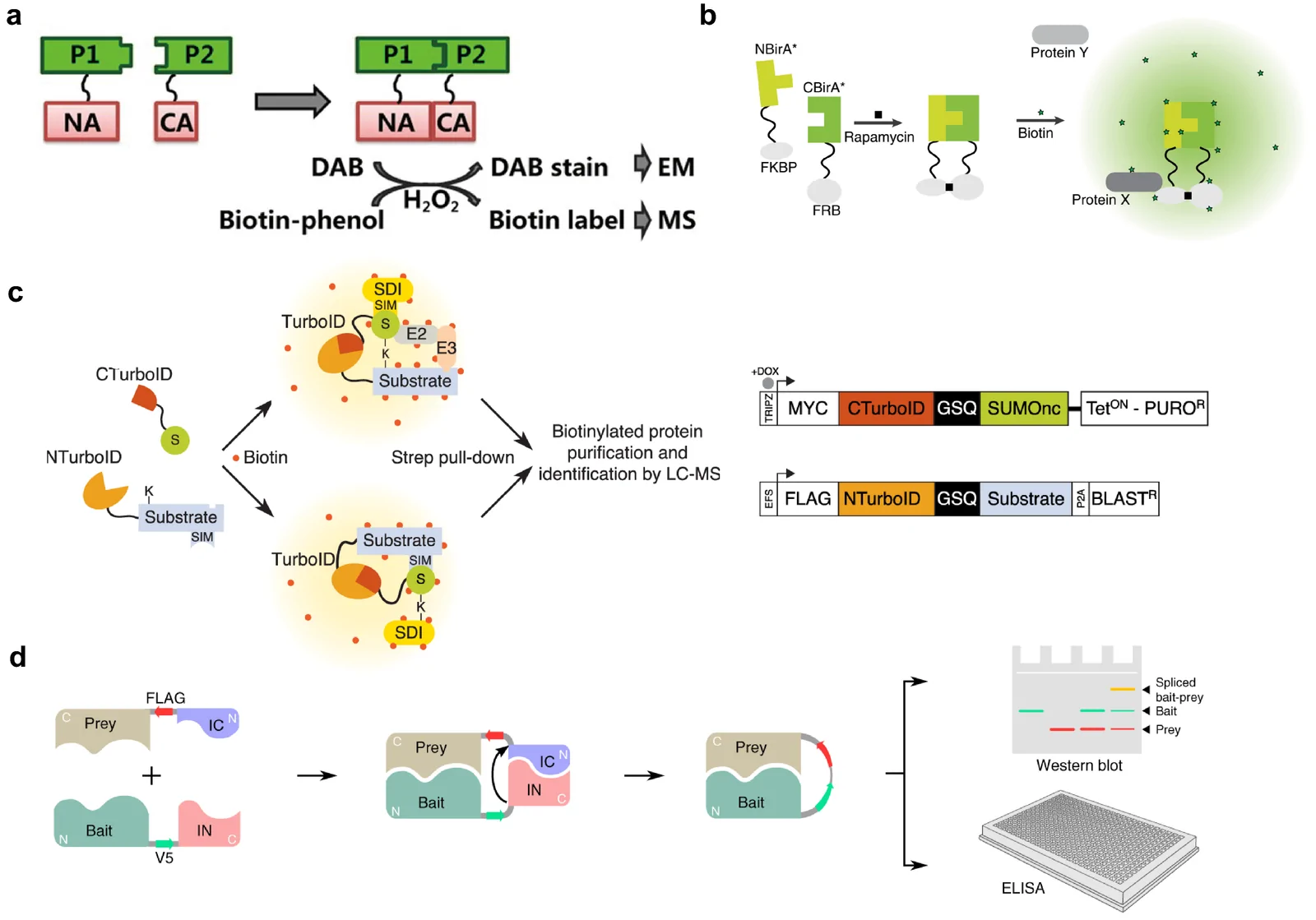
Split-complementation and split-protein recording strategies. (**a**) Interaction between proteins P1 and P2 juxtaposes the N- and C-terminal split engineered ascorbate peroxidase (APEX) fragments, restoring 3,3′-diaminobenzidine (DAB)/$H_{2}$$O_{2}$ staining for electron microscopy or biotin–phenol/$H_{2}$$O_{2}$ labeling for mass spectrometry. (**b**) Rapamycin-induced FK506-binding protein (FKBP)/FKBP–rapamycin-binding (FRB) colocalization reconstitutes NBirA* and CBirA*, producing local biotinylation around Protein X, Protein Y, and neighboring targets. (**c**) SUMO-ID reconstitutes split-TurboID when a substrate is SUMOylated or engages a SUMO-dependent interactor, enabling biotinylation followed by streptavidin enrichment and liquid chromatography–mass spectrometry; the inducible fusion constructs used in the system are shown on the right. (**d**) In split intein-mediated protein ligation (SIMPL), bait–prey association reconstitutes a split intein, ligates the two partners, and generates a product detectable by Western blotting or an enzyme-linked immunosorbent assay. Open-license credits for the adapted panels: (**a**) Xue et al., Figure 1a, Ref. [42]; (**b**) Schopp et al., Figure 1a, Ref. [22]; (**c**) Barroso-Gomila et al., Figure 1a,b, Ref. [51]; (**d**) Yao et al., Figure 1a, Ref. [52]. Panels (**a**–**d**) were cropped, resized, relabeled, and recomposed from sources licensed under CC BY 4.0; https://creativecommons.org/licenses/by/4.0/ (accessed on 12 October 2026).

In 2016, Martell et al. systematically screened 17 candidate cleavage sites in horseradish peroxidase (HRP) and combined this process with directed evolution to construct split-HRP (sHRP) [24]. When the optimized N- and C-terminal fragments were fused to a pair of trans-synaptic interacting proteins, their interaction brought the HRP fragments together in the synaptic cleft and restored peroxidase activity. In the presence of biotin–phenol and hydrogen peroxide, proteins near the contact region were labeled. This study provided the first application of split PL to the detection of intercellular interactions [24]. The restricted catalytic activity of HRP in reducing intracellular environments [41], however, prompted the development of enzyme platforms better suited to intracellular applications. In 2019, Han et al. used directed evolution to develop split engineered ascorbate peroxidase 2 (split-APEX2), in which APEX2 was divided into a 200-amino-acid N-terminal AP fragment and a 50-amino-acid C-terminal EX fragment [25]. Interaction-driven assembly of these fragments restored peroxidase activity in the cytosol and enabled PL at mitochondria–endoplasmic reticulum contact sites [25]. Separately, Xue et al. optimized an interaction-dependent split-APEX complementation system and used it to identify key interaction regions in the stromal interaction molecule 1 (STIM1)–Orai1 complex (Figure 1a) [42]. Split-APEX2 and related systems have also been used to examine the spatial organization of the Ebola virus polymerase complex [43] and perform spatial mapping and interactome profiling of $N^{6}$-methyladenosine-modified R-loops [44].

For biotin ligases, Schopp et al. systematically screened split sites in BirA* and evaluated fragment complementation using the FK506-binding protein (FKBP)/FKBP–rapamycin-binding (FRB) rapamycin-inducible dimerization system. E256/G257 was identified as the optimal split site, establishing split-BioID (Figure 1b) [22]. Split-BioID can validate a binary PPI while identifying candidate proteins near the reconstructed complex without prespecifying them. The slow labeling kinetics of BioID, which require several hours, nevertheless limit its ability to capture transient interactions [14,45]. Cho et al. subsequently used the SPELL algorithm to predict candidate cleavage sites and experimentally screened 14 positions, identifying L73/G74 as a low-affinity split site suitable for conditional reconstitution and creating split-TurboID [23,46]. Intact TurboID labels much more rapidly than BioID and can generate effective labeling within approximately 10 min. In the original split-TurboID study, labeling became detectable after 30 min in the rapamycin-induced fragment-complementation assay, whereas proteomic experiments used longer labeling periods. The approximately 10 min window of intact TurboID should therefore not be directly assigned to split-TurboID [14,23]. Applied to endoplasmic reticulum–mitochondria contact sites, split-TurboID identified more than 100 endogenous proteins [23]. The platform was later extended to in vivo labeling at neuron–glia interfaces [47,48] and to mapping defined protein-complex networks in plant and mammalian systems [49,50]. SUMO-ID applies split-TurboID to small ubiquitin-like modifier (SUMO) signaling (Figure 1c) [51].

An alternative approach uses a split ligation protein rather than a split catalytic enzyme. Split intein-mediated protein ligation (SIMPL) converts bait–prey association into a covalently linked product (Figure 1d) [52]. In the SMART–SpyCatcher system, SpyCatcher003 is divided into SpyN and SpyC fragments, which are fused to molecules targeting different cell-surface antigens, such as human epidermal growth factor receptor 2 (HER2) and epidermal growth factor receptor (EGFR) [53]. When both fragments bind the same cell surface, proximity triggers conditional protein trans-splicing and reconstructs functional SpyCatcher. The reconstituted protein then covalently captures a SpyTag003-bearing catalytic reporter, such as SpyTag003–APEX2 or a SpyTag003-conjugated iridium photocatalyst. Hydrogen peroxide or blue light activates the recruited catalytic module and initiates PL on double-positive cells. Thus, unlike systems in which the catalytic enzyme itself is split, SMART–SpyCatcher divides a ligation module and uses its reconstitution to recruit the downstream catalyst.

## 3. Interface-Localized Labeling of Intercellular Interactions

Physical interactions between cells are central to immune responses, tissue development, neuronal communication, and the tumor microenvironment. Conventional methods, however, have difficulty recording both the identities and molecular features of interacting cells in situ [21,26]. Interface-localized PL strategies anchor a labeling enzyme or catalytic module to the cell surface and generate a localized signal at the contact interface, enabling physical intercellular interactions to be recorded and analyzed [54].

Pasqual et al. reported labeling immune partnerships by SorTagging intercellular contacts (LIPSTIC), which uses a Staphylococcus aureus sortase A (SrtA)-mediated transpeptidation reaction to transfer a labeling molecule carrying an LPETG sequence to a G5 acceptor on the adjacent cell surface [26]. When SrtA and G5 are displayed on the surfaces of two different cell types, contact between the cells results in contact-dependent covalent labeling. In living mice, LIPSTIC recorded dynamic “kiss-and-run” interactions between T cells and dendritic cells. Combined with flow cytometry, it identified interacting cell populations and revealed antigen-dependent interaction patterns at different stages of T-cell priming [26]. Ge et al. subsequently developed enzyme-mediated intercellular proximity labeling (EXCELL), in which the engineered sortase A variant mgSrtA recognizes endogenous proteins bearing an N-terminal monoglycine. EXCELL therefore performs intercellular labeling without prior installation of a G5 acceptor and expands the range of settings in which cell–cell interactions can be detected [27].

**Figure 2:**
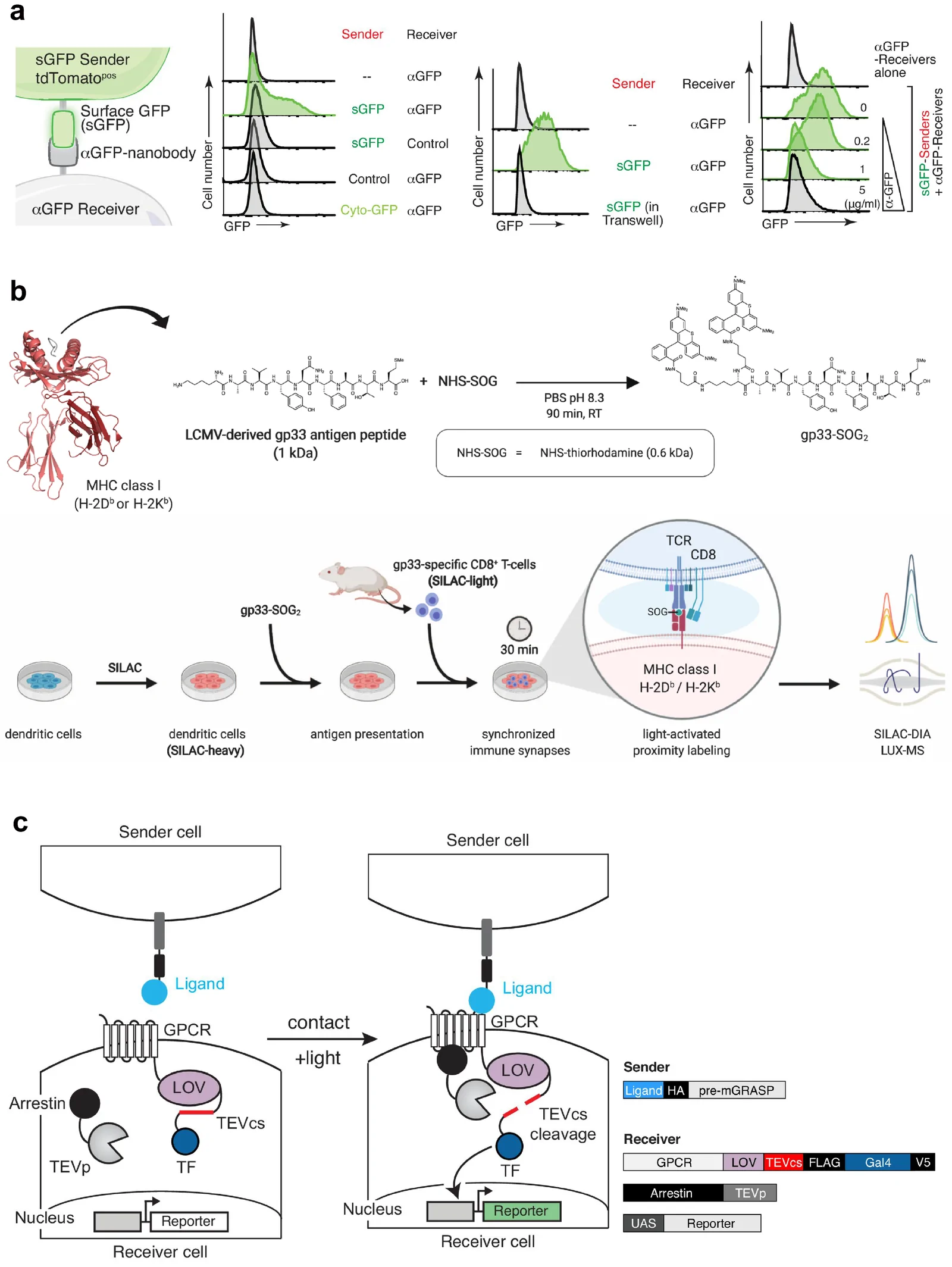
Contact-dependent transfer, photocatalytic mapping, and transcriptional recording of intercellular interactions. (**a**) In G-baToN, surface green fluorescent protein (GFP) on sender cells is captured by an anti-GFP nanobody on receiver cells during direct contact; the flow-cytometry profiles distinguish contact-dependent transfer from receiver-only, cytosolic-GFP, and Transwell controls and show competition by soluble anti-GFP. (**b**) LUX-MS couples the gp33 antigenic peptide to a singlet-oxygen generator and uses light-activated proximity labeling at synchronized gp33-specific dendritic-cell/${\mathrm{CD8}}^{+}$ T-cell immunosynapses before stable isotope labeling by amino acids in cell culture–data-independent acquisition (SILAC–DIA) mass spectrometry. (**c**) TRACC requires both ligand–G-protein-coupled receptor (GPCR) contact and light: contact recruits arrestin–tobacco etch virus (TEV) protease, light uncages the TEV cleavage site, and the released transcription factor activates a reporter in the receiver cell. Open-license credits for the adapted panels: (**a**) Tang et al., Figure 1A–D, Ref. [55]; (**b**) Müller et al., Figure 6a,b, Ref. [56]; (**c**) Cho et al., Figure 1A,B, Ref. [57]. Panels (**a**–**c**) were cropped, resized, relabeled, and recomposed from sources licensed under CC BY 4.0; https://creativecommons.org/licenses/by/4.0/ (accessed on 12 October 2026).

Unlike LIPSTIC and EXCELL, which are based on sortase-mediated transfer, FucoID uses fucosyltransferase-mediated glycosylation to transfer a biotinylated fucose group from a labeling cell to glycoproteins on an adjacent cell surface [28]. Anchoring the enzyme to dendritic cells enabled contact-dependent labeling during recognition by antigen-specific T cells and identification of tumor antigen-reactive ${\mathrm{CD4}}^{+}$ and ${\mathrm{CD8}}^{+}$ T cells from mixed populations without prior specification of the antigen [28]. FucoID further showed that the labeled T cells possessed distinct T-cell receptor (TCR) clonotypes and transcriptional features, providing a route for rapidly identifying antigen-reactive T cells in cancer immunotherapy [28]. FucoID requires direct cell contact, accessible cell-surface N-acetyllactosamine acceptor glycans, surface-bound fucosyltransferase, and a modified GDP–fucose donor. Its labels identify contacting cells but do not establish a complete protein interactome or a specific receptor–ligand pair [28,54]. The open-license examples in Figure 2 illustrate complementary ways of recording the same biological event: G-baToN transfers a fluorescent surface marker, LUX-MS maps proteins at an illuminated immune synapse, and TRACC converts direct contact into a light-gated transcriptional output [55,56,57].

## 4. Light-Controlled Proximity Labeling with Spatiotemporal Resolution

In split-complementation strategies, conditional PL is initiated when two target molecules physically contact one another or reach a distance that permits fragment complementation. These systems therefore primarily provide event-dependent spatial selectivity; their effective temporal resolution also depends on fragment reconstitution, substrate delivery, and catalytic kinetics. Biological processes such as dynamic signal transduction, transient protein trafficking between organelles, and time-dependent reorganization of molecular networks under stress require not only localization of the event but also definition of its temporal window [15,29,58].

**Figure 3:**
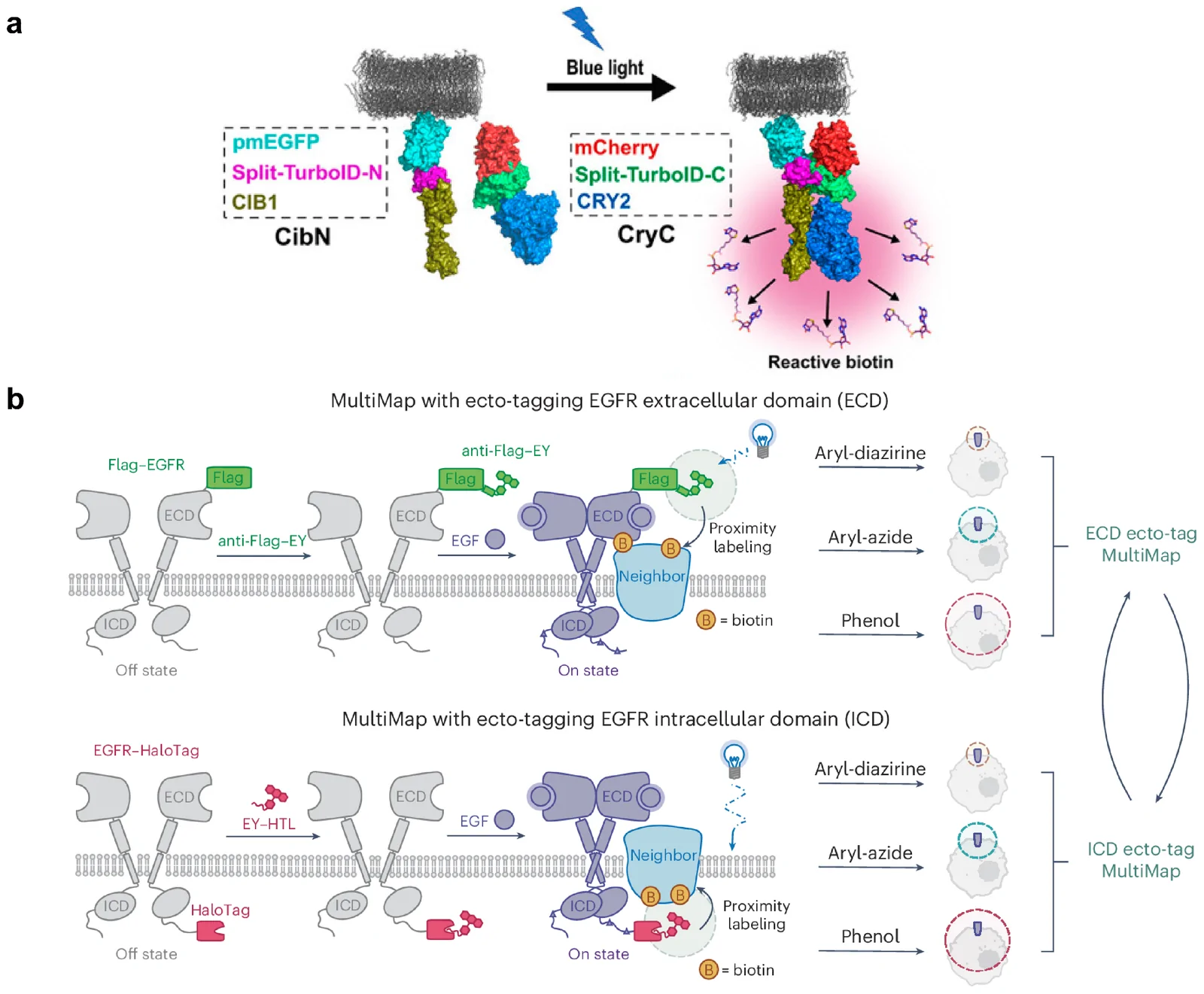
Representative mechanisms of light-controlled proximity labeling. (**a**) Light-activated BioID (LAB) fuses split-TurboID fragments to the blue-light-responsive CIB1 and CRY2 modules. Blue light recruits cytosolic CryC to membrane-localized CibN, reconstitutes split-TurboID, and initiates proximity biotinylation. (**b**) MultiMap targets Eosin Y towards engineered Flag-tagged epidermal growth factor receptor (EGFR) extracellular domains or HaloTag-fused EGFR intracellular domains. Illumination activates aryl-diazirine, aryl-azide, and phenol probes to obtain EGFR ecto-tag maps across different labeling ranges. Open-license credits for the adapted panels: (**a**) Shafraz et al., Figure 1A, Ref. [59]; (**b**) Lin et al., Figure 1a, Ref. [38]. Panels (**a**,**b**) were cropped, resized, relabeled, and recomposed from sources licensed under CC BY 4.0; https://creativecommons.org/licenses/by/4.0/ (accessed on 12 October 2026).

One route to temporal control is to introduce a photosensitive domain into a PL enzyme so that illumination-induced conformational changes regulate catalytic activity. Lee et al. combined structure-guided screening and directed evolution to integrate a light–oxygen–voltage (LOV) domain into TurboID, generating LOV-Turbo [29]. In darkness, conformational restraint imposed by the LOV domain suppresses TurboID activity. Blue-light illumination changes the LOV conformation and releases this inhibition, activating labeling; after illumination ceases, the domain returns to its dark state and again inhibits TurboID. This reversible regulation controls the onset and termination of labeling and substantially reduces background accumulated during prolonged TurboID incubation, particularly in biotin-rich environments such as neurons [29]. LOV-Turbo can also be activated through luciferase-mediated bioluminescence resonance energy transfer (BRET) rather than an external light source. This mode provides a proof of concept for coupling labeling to assembly of a defined protein complex or occurrence of a biological event, although its energy-transfer efficiency and range of application require further optimization [29]. LOV-Turbo has been validated in multiple cell types and organelles, and pulse–chase labeling has been used to identify proteins that move among the endoplasmic reticulum, nucleus, and mitochondria during cellular stress [29]. A complementary design, light-activated BioID (LAB), uses blue-light-induced CIB1–CRY2 association to reconstitute split-TurboID, thereby coupling illumination to enzyme assembly rather than directly regulating the catalytic domain (Figure 3a) [59].

Photocatalytic proximity labeling (PPL) differs from strategies in which photosensitive domains regulate protein function or enzyme activity. PPL uses a photocatalyst to generate reactive intermediates directly under illumination and thereby covalently label neighboring molecules [60]. Transition-metal complexes and organic chromophores can serve as small-molecule photocatalysts and can be targeted to a region of interest by chemical conjugation or antibodies without genetic manipulation [60]. Genetically encoded flavoprotein photocatalysts can instead be fused to the target and activated by light, without delivering an external catalyst [35,36,61].

Mini singlet oxygen generator (miniSOG)-based photoactivation-dependent proximity labeling (PDPL) and reactive oxygen species-induced labeling and identification (RinID) use blue light to generate reactive oxygen species. Amine probes capture nearby oxidized proteins for enrichment and mass spectrometry [35,61]. RinID uses minute-scale illumination. Light-induced interactome tagging (LITag) uses an engineered light–oxygen–voltage (LOV*) flavoprotein with biotin–phenol or biotin–aniline probes to label nearby proteins within seconds of illumination [36]. Directed evolution of miniSOG yielded Lantern, which labels RNA and proteins in separate sub-minute assays. It supports chromophore-assisted proximity labeling for transcriptomic (CAP-seq), proteomic (CAP-MS), and cell-surface (CAP-CELL) analyses [37]. Labeling times depend on the probe, illumination conditions, and target biomolecules [35,36,37].

Qiu et al. anchored the ${\mathrm{Ru(bpy)}}_{3}^{2+}$ photocatalyst to the cell surface to establish a PPL system [62]. Upon visible-light excitation, ${\mathrm{Ru(bpy)}}_{3}^{2+}$ generates reactive intermediates through energy- or electron-transfer pathways, enabling covalent labeling of nearby proteins. The singlet-oxygen pathway provided higher labeling efficiency and specificity than the single-electron-transfer pathway [62]. Because the catalytic reaction can be defined by illumination, the system labels cell-contact regions within selected temporal windows and supports spatiotemporally resolved detection of physical interactions between cells. The strategy was further deployed on the surface of TCR-engineered T cells. TCR-mediated antigen-specific recognition captured different antigen-presenting cells, while light-controlled PL resolved differences in cell contact associated with antigen affinity, offering a genetically noninvasive approach for studying heterogeneity among antigen-presenting cells [62].

Lin et al. developed MultiMap, which uses a single organic photocatalyst, Eosin Y, to activate three classes of photoprobes—diazirine, aryl azide, and phenol—with probe-dependent labeling ranges [30]. Eosin Y was anchored to either the extracellular or intracellular domain of EGFR (Figure 3b) [38], and labeling was initiated by illumination at different times after ligand activation. This design captured changes in the EGFR-proximal proteome from several minutes to one hour and delineated networks associated with different phases of signaling. MultiMap was also extended to intercellular interactions and used to profile four distinct intercellular synapses induced by bispecific T-cell engagers and chimeric antigen receptors (CARs) [30]. Recent studies also used an aggregation-induced emission luminogen to label the Golgi-associated secretome and HisTag–iridium coordination to enable cell-surface labeling and contact mapping [63,64].

Lou et al. reported CAT-Tissue, which extended PPL from cultured cells to primary tissue sections. A nanobody–chlorin e6 conjugate served as the photocatalyst and biotin–aniline as the probe, enabling in situ analysis of tumor–immune interactions under deep-red illumination [39]. CAT-Tissue is a non-genetically encoded labeling platform. In combination with bulk RNA sequencing, it revealed distinct gene-expression patterns between tumor-proximal and tumor-distal lymphocytes; tumor-proximal ${\mathrm{CD8}}^{+}$ T cells displayed heterogeneous activation, effector, and exhaustion phenotypes [39].

## 5. Spatial-Information Decoding and Dynamic Tracking Through Multiple Labeling Reactions

Multiple-labeling strategies are designed to resolve molecular processes that evolve over time, including directional protein trafficking between subcellular compartments, changes in the spatial relationships of organelle-contact regions, and identification of intermediary molecules in complex interaction networks. These strategies use multiple labels in different ways. TransitID encodes trafficking history between source and destination compartments as dual labeling; OrthoID uses orthogonal chemical tags to distinguish spatial origin and contact interfaces; and STUPPIT and two-level spatially localized proximity labeling (P2L) implement sequential or hierarchical reactions for multicontext logic filtering. Multiple labeling can therefore track dynamic trafficking, distinguish spatial origins, or screen key intermediaries in complex networks [15,31].

**Figure 4:**
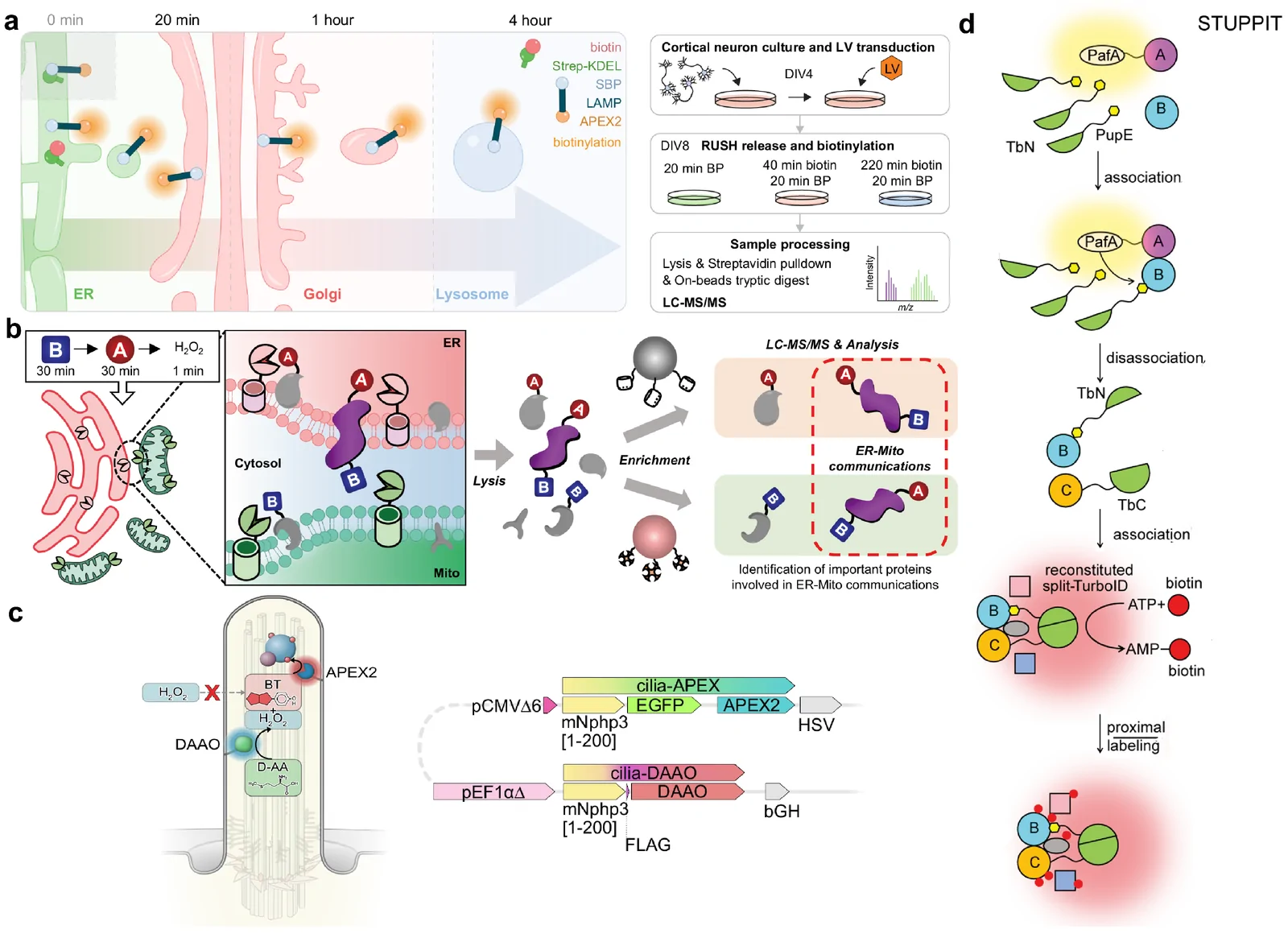
Spatial-information decoding and dynamic tracking through conditional and multiple labeling reactions. (**a**) POTATOMap combines the retention using selective hooks (RUSH) system with lysosome-associated membrane protein 1 (LAMP1)–APEX2 to synchronize cargo release from the endoplasmic reticulum and perform proximity biotinylation at successive Golgi and lysosomal trafficking stages before streptavidin enrichment and liquid chromatography–tandem mass spectrometry. (**b**) OrthoID uses orthogonal TurboID biotinylation and APEX2 adamantylation to distinguish endoplasmic reticulum (ER), mitochondrial, and ER–mitochondria-contact proteins after parallel enrichment and LC–MS/MS. (**c**) In situ APEX activation (iAPEX) targets D-amino acid oxidase and APEX2 towards primary cilia; local hydrogen-peroxide production from a D-amino acid activates nearby APEX2 while avoiding externally supplied $H_{2}$$O_{2}$. (**d**) STUPPIT first uses pupylation-based interaction tagging (PUP-IT) to install TbN–PupE on proteins near an upstream protein. Association of a tagged intermediary with a TbC-bearing downstream protein reconstitutes split-TurboID and drives a second biotinylation step. Open-license credits for the adapted panels: (**a**) Li et al., Figure 2a,b, Ref. [65]; (**b**) Lee et al., Figure 1, Ref. [33]; (**c**) Sroka et al., Figure 1c,d, Ref. [66]; (**d**) Xie et al., Figure 1C, Ref. [34]. Panels (**a**–**d**) were cropped, resized, relabeled, and recomposed from sources licensed under CC BY 4.0; https://creativecommons.org/licenses/by/4.0/ (accessed on 12 October 2026).

TransitID, reported by Qin et al., is a representative multiple-labeling method for tracking dynamic protein trafficking [32]. Two orthogonal PL enzymes, TurboID and APEX2, are targeted towards “source” and “destination” compartments, respectively. TurboID labels proteins in the source compartment using biotin, whereas APEX2 labels proteins in the destination compartment using alkyne–phenol. Mass-spectrometric identification of proteins bearing both biotin and alkyne tags provides a systematic readout of endogenous proteins that have undergone trafficking. TransitID has mapped protein transport between the cytosol and mitochondria, between the cytosol and nucleus, and between the nucleolus and stress granules [32]. In the stress-granule setting, TransitID has shown that stress granules protect the transcription factor JUN during oxidative stress. It has also identified proteins transferred between macrophages and cancer cells [32]. POTATOMap instead combines RUSH-mediated synchronization with APEX2 labeling to obtain time-resolved proximity snapshots as LAMP1 traffics from the endoplasmic reticulum through the Golgi to lysosomes (Figure 4a) [65].

OrthoID illustrates the use of orthogonal multiple labeling at organelle contact sites (Figure 4b) [33]. TurboID and APEX2 are targeted to the mitochondrial outer membrane and endoplasmic-reticulum membrane, respectively, and introduce orthogonally distinguishable chemical tags: TurboID produces a biotin signal, whereas an APEX2-mediated phenolic reaction introduces an adamantane tag. The combined information distinguishes proteins according to their organelle of origin and identifies proteins at the contact interface. Applied to the proteome of mitochondria-associated endoplasmic-reticulum membranes (MAMs), OrthoID identified candidate regulators including LRC59 and characterized dynamic changes in MAM protein composition during mitophagy [33].

Xie et al. reported STUPPIT, which couples pupylation-based interaction tagging (PUP-IT) to split-TurboID to identify intermediary molecules that bridge two proteins lacking a direct interaction (Figure 4d) [34]. PUP-IT first attaches a TbN–PupE tag to proteins near an upstream protein. If one of these intermediaries subsequently interacts with a downstream protein carrying TbC, split-TurboID is reconstituted and biotinylates the intermediary. Through two orthogonal enzymatic reactions, STUPPIT selectively captures bridging molecules in protein-interaction networks. The method was validated with several known protein triads, and proteomic analysis identified candidate intermediary proteins including ERC1 and USP7; both interacted with the key Wnt- and bone morphogenetic protein (BMP)-pathway components β-catenin and SMAD4 [34].

Chen et al. extended enzyme cascades to cross-hierarchy spatial encoding through P2L [20]. The method couples galactose oxidase (GAO)-mediated glycan oxidation to HRP-mediated PL. GAO generates hydrogen peroxide locally on the target cell surface, which activates HRP directed to a specified glycan structure and triggers protein labeling. Because the reaction depends jointly on target-cell identity and a defined glycan modification, P2L implements two-condition spatial logic gating. It distinguishes target cells with different glycosylation features within mixed populations and supports covalent assembly at selected cell-surface sites [20]. This design extends the spatial selectivity of PL by combining biological information across hierarchical levels to identify defined cell populations and glycosylation microenvironments. In situ APEX activation (iAPEX) uses locally targeted D-amino acid oxidase to generate the hydrogen peroxide required by nearby APEX2, adding an enzymatic activation gate rather than a second labeling tag (Figure 4c) [66].

## 6. Summary and Outlook

Advances in PL have shifted in situ studies of molecular interactions from analysis based on a single spatial localization toward the interrogation of condition-dependent events [15,67]. By incorporating split complementation, interface localization of catalytic modules, light-based regulation, and multiple labeling reactions, conditional PL can respond to defined molecular interactions, cell contacts, or external stimuli and thereby broaden the use of conventional PL in complex biological systems [31,67]. Table 1 summarizes these differences to help readers choose a method for the required readout, time window, and biological setting. Their gains in selectivity depend on enzyme-reconstitution efficiency, the triggering mechanism, and the specific application context, while their outputs expand access to dynamic molecular processes and complex spatial relationships.

Conditional PL nevertheless faces challenges in sensitivity, generalizability, and system compatibility [45,68]. Split systems depend on effective reconstitution of two enzyme fragments, so labeling efficiency and response speed are influenced by fragment design and the expression environment [23,25]. Interface-localized strategies can record intercellular contact events, but some require cell-surface engineering, limiting their deployment in complex biological systems [69,70]. Light-controlled PL provides time-resolved reaction control, although illumination conditions, tissue penetration, and light-induced side reactions must be considered in further applications [71,72]. For multiple-labeling strategies, the orthogonality, reaction compatibility, and analytical interpretation of different labeling systems still require optimization [33,67].

Further development of conditional PL will depend on a broader range of catalytic systems and more precise triggering modes [73]. New labeling enzymes, chemical probes, and photosensitive modules could expand the conditions that can be encoded [73,74]. Integration with single-cell analysis, spatial omics, and high-resolution imaging may connect proximity records to cellular heterogeneity and tissue architecture [73,75,76]. Comparable analytical frameworks across platforms and improved compatibility with native tissues and animal models are also needed [15,58,76].

Across these designs, molecular proximity, cell contact, illumination, and sequential labeling history are converted into covalent records that can be recovered after the event. The biological meaning of those records remains tied to the conditional input, labeling chemistry, and validation strategy used in each system.

## Data Availability

No new data were created or analyzed in this study.
